# Periplasmic phosphorylation of lipid A is linked to the synthesis of undecaprenyl phosphate

**DOI:** 10.1111/j.1365-2958.2007.06044.x

**Published:** 2007-11-02

**Authors:** Thierry Touzé, An X Tran, Jessica V Hankins, Dominique Mengin-Lecreulx, M Stephen Trent

**Affiliations:** 1 Laboratoire des Enveloppes Bactériennes et Antibiotiques, Unité Mixte de Recherche 8619 CNRS, Université Paris-Sud 91405 Orsay, France.; 2 Department of Microbiology, James H. Quillen College of Medicine, East Tennessee State University Johnson City, TN 37614, USA.; 3 Department of Biochemistry and Molecular Biology, Medical College of Georgia Augusta, GA 30912, USA.

## Abstract

One-third of the lipid A found in the *Escherichia coli* outer membrane contains an unsubstituted diphosphate unit at position 1 (lipid A 1-diphosphate). We now report an inner membrane enzyme, LpxT (YeiU), which specifically transfers a phosphate group to lipid A, forming the 1-diphosphate species. ^32^P-labelled lipid A obtained from *lpxT* mutants do not produce lipid A 1-diphosphate. *In vitro* assays with Kdo_2_-[4′-^32^P]lipid A as the acceptor shows that LpxT uses undecaprenyl pyrophosphate as the substrate donor. Inhibition of lipid A 1-diphosphate formation in wild-type bacteria was demonstrated by sequestering undecaprenyl pyrophosphate with the cyclic polypeptide antibiotic bacitracin, providing evidence that undecaprenyl pyrophosphate serves as the donor substrate within whole bacteria. LpxT-catalysed phosphorylation is dependent upon transport of lipid A across the inner membrane by MsbA, a lipid A flippase, indicating a periplasmic active site. In conclusion, we demonstrate a novel pathway in the periplasmic modification of lipid A that is directly linked to the synthesis of undecaprenyl phosphate, an essential carrier lipid required for the synthesis of various bacterial polymers, such as peptidoglycan.

## Introduction

The Gram-negative bacterial cell envelope consists of an inner membrane, an outer membrane and the periplasmic region (Nikaido, 2003). The outer membrane is an asymmetric lipid bilayer with phospholipids forming the inner leaflet and lipopolysaccharides (LPS) forming the outer leaflet (Nikaido, 2003), whereas the inner cytoplasmic membrane is composed of phospholipids. Within the periplasm resides a continuous cross-linked carbohydrate polymer that forms a homogeneous layer outside the cytoplasmic membrane, known as the peptidoglycan layer (Schleifer and Kandler, 1972; van Heijenoort, 2001a). Both LPS and peptidoglycan are essential for maintaining the structural integrity of the Gram-negative cell envelope, and are generally required for viability. LPS acts as an efficient permeability barrier against toxic compounds, such as antibacterial agents and detergents (Raetz and Whitfield, 2002), and peptidoglycan is important for both the shape of the bacterium and protection against internal osmotic pressure (Nanninga, 1998; van Heijenoort, 2001b).

**Fig. 1:**
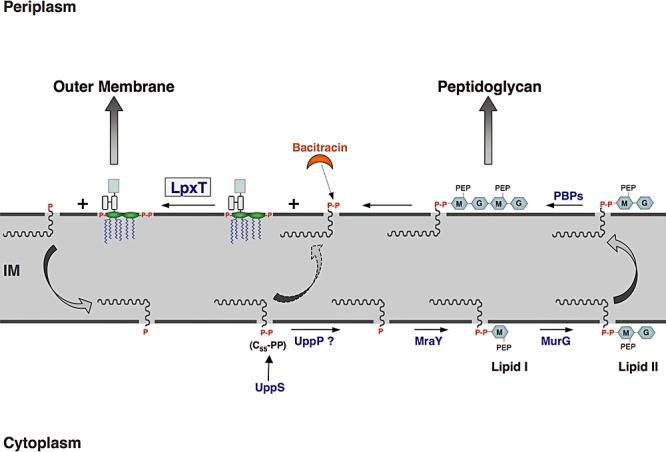
Phosphorylation of lipid A by LpxT is linked to the recycling of C_55_-P. The carrier lipid C_55_-P is first synthesized in its pyrophosphate form (C_55_-PP) by UppS synthase on the cytoplasmic side of the inner membrane. Prior to its use, the lipid must be dephosphorylated by an undecaprenyl pyrophosphatase. An example of the recycling of C_55_-P during the synthesis of the essential bacterial polymer peptidoglycan is shown. The MraY and MurG enzymes catalyse the successive transfers of the MurNAc-pentapeptide and GlcNAc motifs from the peptidoglycan nucleotide precursors onto C_55_-P, generating the lipid I and lipid II intermediates respectively. Following transport of lipid II across the inner membrane, reactions catalysed by the penicillin-binding proteins (PBPs) begin. This results in the generation of free C_55_-PP that must be dephosphorylated prior to its reuse for *de novo* peptidoglycan biosynthesis (van Heijenoort, 2001a, b). LpxT (formerly YeiU) dephosphorylates C_55_-PP and transfers the phosphate group to lipid A to form lipid A 1-diphosphate contributing to the recycling of C_55_-P. It cannot be excluded that LpxT may dephosphorylate C_55_-PP that has been directly transported across the inner membrane (indicated by dashed arrow) as part of the C_55_-P *de novo* synthesis pathway. All enzymes are indicated in blue.

Biosynthesis of LPS and peptidoglycan, as well as other bacterial cell wall polymers, requires an essential carrier lipid, undecaprenyl phosphate (C_55_-P) (Fig. 1). C_55_-P is generated initially from the dephosphorylation of undecaprenyl pyrophosphate (C_55_-PP), which is synthesized in the cytoplasm by UppS, a *cis*-prenyl-pyrophosphate synthase. UppS catalyses the addition of eight isoprene units to farnesyl pyrophosphate (FPP) to form C_55_-PP (Apfel *et al*., 1999; Kato *et al*., 1999). The C_55_-P carries various hydrophilic precursors in a pyrophosphate linkage (C_55_-PP-substrate) across the hydrophobic inner membrane serving as the donor substrate for various biosynthetic pathways within the periplasm. Following the polymerization reaction, the carrier lipid is released in its pyrophosphate form (C_55_-PP), requiring a second dephosphorylation event before the lipid can once again serve as a carrier molecule. An example of C_55_-P synthesis during the biosynthesis of peptidoglycan is shown in Fig. 1 (van Heijenoort, 2001a).

Recently, several inner membrane proteins demonstrating undecaprenyl pryophosphate phosphatase activity were identified in *Escherichia coli* (El Ghachi *et al*., 2004; 2005): BacA and three members from the phosphatidic acid-phosphatase superfamily of phosphatases (PgpB, YbjG and YeiU). Overproduction of the first, BacA (UppP, C_55_-PP phosphatase), has been shown to increase resistance to the cyclic polypeptide antibiotic bacitracin (Bernard *et al*., 2005). Bacitracin strongly binds to C_55_-PP, inhibiting its dephosphorylation to C_55_-P, thereby preventing synthesis of the essential carrier lipid. Presumably, overexpression of BacA results in an increase in the cellular pool of C_55_-P, thus overcoming the effects of the antibiotic. The second, PgpB, was previously identified as a phosphatidylglycerol phosphate phosphatase (Icho and Raetz, 1983) in the synthesis of phosphatidylglycerol, but also displays significant UppP activity *in vitro*. YbjG and YeiU had not been previously characterized.

Overexpression of BacA, PgpB or YbjG results in bacitracin resistance in whole cells, and increases the level of UppP activity in cellular extracts (El Ghachi *et al*., 2005). Interestingly, deletion of both *ybjG* and *pgpB* is required to abolish the growth of a *bacA* mutant. Complementation of a temperature-sensitive triple mutant harbouring deletions in *bacA*, *ybjG* and *pgpB* can be achieved with an intact chromosomal copy of only one of the *bacA*, *ybjG* and *pgpB* genes (El Ghachi *et al*., 2005). YeiU also exhibits UppP activity *in vitro*; however, its expression was not able to complement the triple mutant (El Ghachi *et al*., 2005). This result suggested that YeiU may have other roles besides dephosphorylating C_55_-PP during carrier lipid synthesis.

**Fig. 2:**
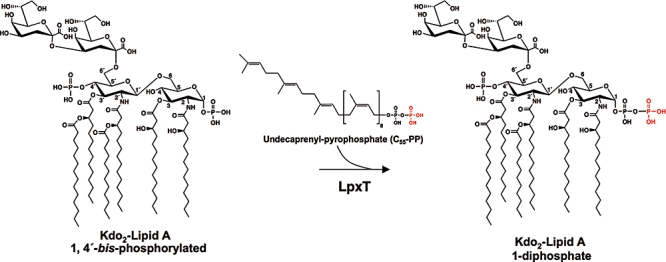
LpxT transfers the distal phosphate from C_55_-PP to the 1-phosphate group of Kdo_2_-lipid A.

We now report that YeiU (renamed LpxT) is involved in the modification of the lipid A domain of Gram-negative LPS. Lipid A serves as the hydrophobic anchor of LPS, and is required to maintain the integrity of the outer membrane barrier (Raetz and Whitfield, 2002). In *E. coli* K-12, lipid A consists of a β(1′-6)-linked disaccharide of glucosamine that is acylated with *R-*3-hydroxymyristate at the 2, 3, 2′ and 3′ positions, and phosphorylated at the 1 and 4′ positions (Raetz and Whitfield, 2002; Trent *et al*., 2006) (Fig. 2). The majority of lipid A isolated from wild-type *E. coli* K-12 contains monophosphate moieties at positions 1 and 4′ (designated lipid A 1-, 4′-*bis*-phosphate). The genes encoding the enzymatic machinery required for the biosynthesis of *bis*-phosphorylated lipid A have been identified. Approximately one-third of the lipid A molecules in the *E. coli* K-12 outer membrane contain a diphosphate unit at the 1 position (termed lipid A 1-diphosphate) (Fig. 2) that originates by an unknown mechanism (Zhou *et al*., 1999). We demonstrate that deletion of *lpxT* results in the production of LPS containing only the *bis*-phosphorylated lipid A species. Purified LpxT uses C_55_-PP as its phosphate donor substrate catalysing the formation of 1-diphosphate lipid A (Figs 1 and 2). Removal and transfer of the phosphate group from C_55_-PP within the bacterial cell is dependent upon MsbA (Doerrler and Raetz, 2002), an essential LPS flippase that transfers the lipid A-core domain to the periplasmic side of the inner membrane prior to addition of O-antigen (Raetz and Whitfield, 2002). However, MsbA is not required for enzymatic function of LpxT during *in vitro* assay. Sequestration of C_55_-PP by exposure of *E. coli* K-12 to levels of bacitracin just below the minimal inhibitory concentration (MIC) inhibited lipid A 1-diphosphate formation in whole bacteria. Collectively, we have identified the last enzyme required for lipid A biosynthesis in *E. coli* K-12, and demonstrated a novel pathway in the synthesis of LPS that is directly linked to the synthesis of C_55_-PP.

## Results

### Formation of lipid A 1-diphosphate in *E. coli* K-12 is catalysed by LpxT

LpxT is predicted to be 249 amino acids long and contains five putative membrane-spanning regions [see http://www.cbs.dtu.dk/services/TMHMM-2.0 (Sonnhammer *et al*., 1998)]. LpxT exhibits sequence similarity to members of the phosphatidic acid-phosphatase family (PAP2) and possesses the common conserved phosphatase motif K*X*_6_RP-(*X*_12-54_)-PSGH-(*X*_31_-_54_)-SRX_5_HX_3_D, previously identified by Stukey and Carman (1997). Further examination of LpxT using the COG (Clusters of Orthologous Groups) database (Wheeler *et al*., 2003) revealed that LpxT is a member of the same COG groups (COG0671) as Hp0021 (LpxE_HP_). LpxE_HP_ was previously identified as a lipid A 1-phosphatase of *Helicobacter pylori* involved in the two-step modification of the 1 position of *H. pylori* lipid A (Tran *et al*., 2004). Like LpxT, LpxE_HP_ is also a member of the PAP2 superfamily of phosphatases. As LpxT was not able to sustain cell growth in the triple mutant BWTsbacA, we wanted to examine another possible role for LpxT involving lipid A modification.

**Fig. 3:**
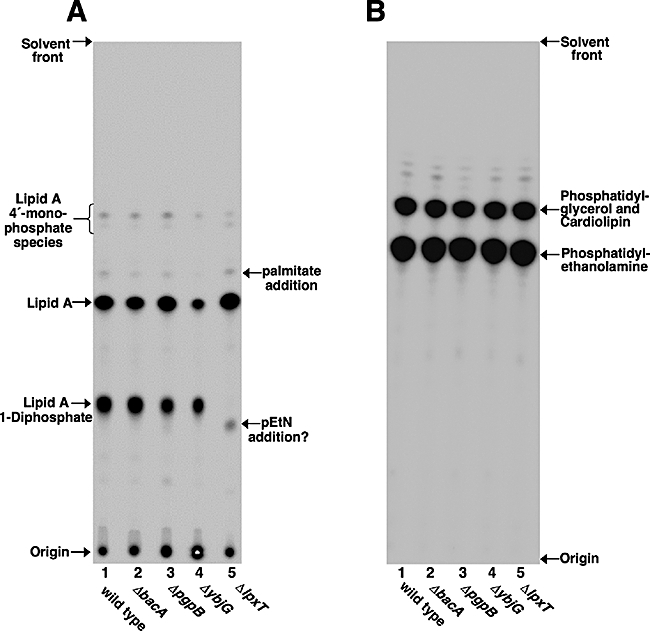
Comparison of ^32^P-labelled lipid A (A) and phospholipid (B) fractions isolated from wild-type BW25113 and UppP-deficient mutants. ^32^P-labelled lipids were isolated as described in *Experimental procedures* section, separated by TLC and visualized by PhosphorImaging. The identities of the major species of lipid A and phospholipids (Zhou *et al*., 1999) are indicated. Minor lipid A species are also indicated. The 4′-monophosphate species arise by the loss of the 1-phosphate group during pH 4.5 hydrolysis at 100°C.

Thin-layer chromatography (TLC) analysis of ^32^P-labelled lipid A species isolated from wild-type *E. coli* strain BW25113 and its isogenic *bacA*, *pgpB* and *ybjG* mutants, previously generated (El Ghachi *et al*., 2005), revealed the two typical lipid A species; hexa-acylated lipid A 1,4′-*bis*-phosphate (lipid A) and hexa-acylated lipid A 1-diphosphate (lipid A 1-diphosphate), which contains a monophosphate group at position 4′ and an unsubstituted diphosphate unit at position 1 (Fig. 3A, lanes 1–4). The ratio of lipid A to lipid A 1-diphosphate was not altered in these mutants when compared with wild type (Fig. 3A, lanes 2–4), thus clearly demonstrating that both BacA and YbjG are not involved in lipid A 1-diphosphate formation or in lipid A modification (Fig. 3A, lanes 2 and 4). Our result corroborates the previous findings of Zhou *et al*. (1999) in that *pgpB*, *E. coli* phosphatidylglycerophosphate phosphatase, does not alter the ratio of lipid A to lipid A 1-diphosphate when compared with wild-type *E. coli* K-12 (Fig. 3A, lane 3). However, to our surprise, deletion of *lpxT* in BW25113 resulted in the total loss of the lipid A 1-diphosphate species (Fig. 3A, lane 5). This result indicates that in *E. coli* K-12 strains lipid A 1-diphosphate formation is dependent on the proper function of LpxT. Deletion of *lpxT* in BW25113 also revealed the presence of a minor lipid species. Based upon the migration of the lipid species and previous studies by Zhou *et al*. (1999), the minor lipid observed in the Δ*lpxT* mutant is the addition of a single phosphoethanolamine (pEtN) unit to lipid A (Fig. 3A, lane 5). Given that LpxT is a member of a larger family of phosphatases shown to play a role in the synthesis of bacterial lipids, the ^32^P-labelled phospholipids of each mutant were compared with that of wild type. Based upon TLC analysis, inactivation of *lpxT* had no effect on synthesis of the major phospholipids in *E. coli* (Fig. 3B).

### LpxT restores 1-phosphotransferase activities in *ΔlpxT*-null mutants

**Fig. 4:**
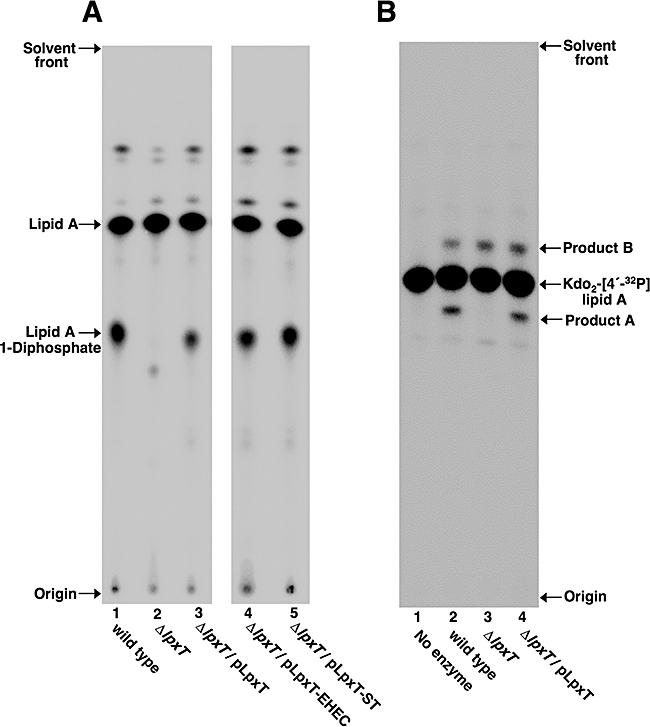
A. Complementation of *lpxT*-deficient mutants with a plasmid expressing LpxT. ^32^P-labelled lipid A species from wild-type BW25113, Δ*lpxT* mutant and Δ*lpxT* mutants expressing the plasmids p*Trc*LpxT, p*Trc*LpxT-EHEC or p*Trc*LpxT-ST were separated by TLC and visualized by PhosphorImaging. The presence of lipid A 1-diphosphate was restored in Δ*lpxT* mutants expressing the plasmids p*Trc*LpxT, p*Trc*LpxT-EHEC or p*Trc*LpxT-ST. B. Enzymatic confirmation of LpxT. Membranes from *E. coli* K-12 strains, BW25113, Δ*lpxT* and Δ*lpxT* expressing p*Trc*LpxT were assayed for 1-phosphotransferase activity using Kdo_2_-[4′-^32^P]lipid A. The protein concentration was 0.5 mg ml^−1^, and assays were carried out for 3 h at 30°C with 5 μM Kdo_2_-[4′-^32^P]lipid A and 100 μM C_55_-PP as the substrates. The reaction products were separated by TLC and detected with PhosphorImager analysis.

To demonstrate the formation of lipid A 1-diphosphate is dependent upon LpxT; the *lpxT* gene was cloned into the plasmid p*Trc*99A and transformed into the *lpxT* mutant, as previously described (El Ghachi *et al*., 2005). TLC analysis of ^32^P-labelled lipid A species isolated from wild-type *E. coli* strain BW25113, BW25113 Δ*lpxT* and BW25113 Δ*lpxT* carrying the plasmid p*Trc*LpxT demonstrated that formation of lipid A 1-diphosphate is dependent upon a functional LpxT (Fig. 4A). Introduction of the vector control, p*Trc*99A, into BW25113 Δ*lpxT* had no effect upon the level of 1-diphosphate species (data not shown).

Modification of the phosphate groups of lipid A with 4-amino-4-deoxy-L-arabinose (L-Ara4N) or pEtN in *E. coli* and *Salmonella typhimurium* is regulated by the two-component regulatory system, PmrA/PmrB (Gunn *et al*., 1998). In both wild-type strains of *E. coli*, O157:H7 and *S. typhimurium*, the phosphate group at the 1 position is predominately modified with a pEtN residue (Zhou *et al*., 2001; Kim *et al*., 2006) and not with an additional phosphate group as previously seen with *E. coli* K-12. Therefore, we wanted to investigate whether the *lpxT* genes from both *E. coli* O157:H7 and *S. typhimurium* are functional by heterologously expressing the LpxT protein in an *E. coli* K-12 *lpxT*-null mutant strain, BW25113 Δ*lpxT*. TLC analysis of lipid A species isolated from BW25113 Δ*lpxT* carrying either p*Trc*LpxT-EHEC or p*Trc*LpxT-ST demonstrated that LpxT from either *E. coli* O157:H7 or *S. typhimurium* can successfully complement an *E. coli* K-12 *lpxT* mutant (Fig. 4A, lanes 4 and 5). Although LpxT is overexpressed by the inducible *trc* promoter in these constructs, it is interesting that no more than one-third of the lipid A is found in the diphosphate form.

To confirm the enzymatic function of LpxT *in vitro*, membranes of wild-type *E. coli* K-12 strain BW25113 and BW25113 Δ*lpxT* were isolated and assayed with Kdo_2_-[4′-^32^P]lipid A for 1-phosphotransferase activity. The Kdo sugars are linked to lipid A at the 6′-position and serve as bridge between the lipid A domain and the core oligosaccharide of LPS. Wild-type membranes catalysed the formation of two reaction products (Fig. 4B, lane 2). Product B results from the addition of palmitate by the outer membrane enzyme PagP, which has been thoroughly characterized (Bishop *et al*., 2000). Based upon its migration, Product A arises from conversion of the Kdo_2_-[4′-^32^P]lipid A substrate to a more hydrophilic reaction product. Addition of a phosphate group to the free hydroxyl on the 1-phosphate group of Kdo_2_-[4′-^32^P]lipid A would decrease the hydrophobicity of the lipid substrate, resulting in a slower-migrating lipid species when separated by the employed TLC system (see *Experimental procedures*). Membranes isolated from BW25113 Δ*lpxT* were unable to catalyse the formation of Product A (Fig. 4B, lane 3), but complementation with the covering plasmid (p*Trc*LpxT) restored this activity (lane 4). These data suggest that Product A results from the addition of a phosphate group to lipid A by LpxT. Treatment of the reaction mixture with LpxE, a lipid A 1-phosphatase, resulted in the formation of only 1-dephosphorylated Kdo_2_-[4′-^32^P]lipid A. This demonstrates that phosphate addition by LpxT occurs at the 1 position (Fig. S1). Also, as no exogenous source of phosphate was added to the assay milieu, it suggests that a membrane component serves as the donor substrate. Phosphotransferase activity was also observed when the tetra-acylated lipid A precursor Kdo_2_-[4′-^32^P]lipid IV_A_ was employed as the lipid acceptor, but not with substrates lacking the Kdo sugars (data not shown). The Kdo region has been shown to be required by enzymes involved in the latter steps of lipid A biosynthesis (Clementz *et al*., 1996; Trent *et al*., 2001a; Reynolds *et al*., 2006). Also, it has been shown that mutants of *E. coli* unable to glycosylate their lipid A with Kdo produce a lipid A structure that is tetra-acylated lacking the 1-diphosphate modification (Meredith *et al*., 2006). Divalent cations are not required for LpxT activity, but inhibition can be seen with high concentrations of either CaCl_2_ or MgCl_2_ (≥ 10 mM) (data not shown). The pH optimum of the reaction is 7.0, but significant activity is observed from pH 5.5–7.5 (data not shown).

### LpxT uses C_55_-PP as the phosphate donor

**Fig. 5:**
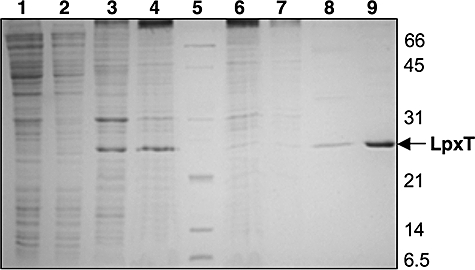
SDS-PAGE of the purified *E. coli* LpxT protein. N-terminal His_6_-tagged LpxT was overproduced in *E. coli* strain CD43(DE3), and purified by affinity chromatography using Ni^2+^-NTA-agarose. Aliquots were loaded on SDS-PAGE and the gel stained with Coomassie Blue. Lane 1: total cell extract; Lane 2: soluble cytosolic fraction; Lane 3: membrane fraction; Lane 4: detergent (DDM) solubilized membrane fraction; Lane 5: molecular weight standards; Lane 6: Ni^2+^-NTA flow through; Lane 7: 10 mM imidazole wash; Lane 8: 30 mM imidazole wash and Lane 9: 400 mM imidazole elution.

**Fig. 6:**
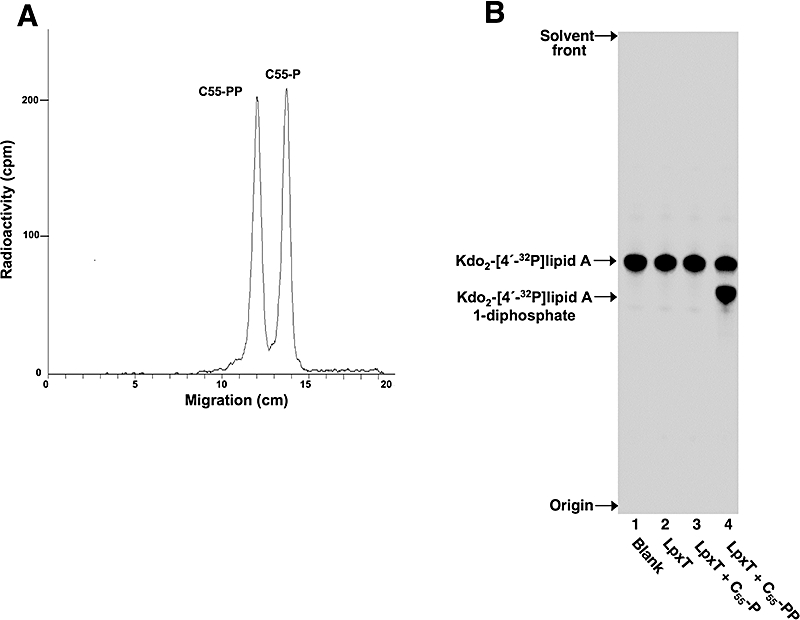
Purified LpxT transfers a phosphate group from C_55_-PP to lipid A. A. Purified LpxT was assayed for phosphatase activity in the presence of [^14^C]C_55_-PP as described in the text. Following separation by TLC, the lipids were located and quantified with a radioactivity scanner. B. Purified LpxT was assayed for 1-phosphotransferase activity using Kdo_2_-[4′-^32^P]lipid A. The protein concentration was 0.001 mg ml^−1^ and assays were carried out for 3 h at 30°C with 5 μM Kdo_2_-[4′-^32^P]lipid A and 100 μM of C_55_-P or C_55_-PP as the substrates. The products were separated by TLC and detected with PhosphorImager analysis.

Previously, El Ghachi *et al*. (2005) demonstrated that membranes isolated from *E. coli* overexpressing LpxT (YeiU) showed a 10-fold increase in the level of C_55_-PP phosphatase activity. Therefore, we wanted to examine if purified LpxT uses C_55_-PP as the phosphate donor during the formation of lipid A 1-diphosphate. To facilitate purification, LpxT was expressed with an N-terminal His_6_-tag in *E. coli* strain C43(DE3) and, as expected, was found to localize to the membrane fraction (Fig. 5, lane 3). Following extraction of the enzyme from membranes using the detergent *n*-dodecyl-β-D-maltoside (DDM) (lane 4), the His_6_-LpxT was purified (lane 9) by affinity chromatography on Ni^2+^-NTA-agarose (see *Experimental procedures*). The purified LpxT was first tested for its ability to dephosphorylate C_55_-PP (Fig. 6A). Pure LpxT clearly exhibited significant C_55_-PP phosphatase activity, with a specific activity at 100 μM C_55_-PP of 0.2 μmol min^−1^ mg^−1^. In contrast, LpxT was not able to dephosphorylate the resulting C_55_-P into C_55_-OH, whatever the amount of C_55_-P formed during the reaction. Importantly, the addition of lipid A acceptor (Kdo_2_-lipid A) did not enhance C_55_-PP phosphatase activity during *in vitro* assay, suggesting that the dephosphorylation and phosphate transfer reactions are uncoupled (data not shown). Next, pure enzyme was assayed with Kdo_2_-[4′-^32^P]lipid A (5 μM) along with either C_55_-P or C_55_-PP (100 μM). As shown in Fig. 6B, lipid A 1-phosphotransferase activity was not detected with purified LpxT alone (lane 2) or with the addition of C_55_-P (lane 3). However, the production of Kdo_2_-[4′-^32^P]lipid A 1-diphosphate was observed upon the addition of C_55_-PP (lane 4). Within the linear range of phosphate transfer, purified LpxT showed a specific activity of 13.7 μmol min^−1^ mg^−1^. The proposed reaction catalysed by LpxT is shown in Fig. 2.

To determine whether other phosphate donors could replace C_55_-PP *in vitro*, we tested several potential high-energy phosphate donors: adenosine 5′-triphosphate (ATP), diacylglycerol pyrophosphate (DGPP), FPP and isopentenyl pyrophosphate (IPP), at various concentrations (0.1–500 μM) for phosphotransferase activity (data not shown). Each potential phosphate donor was assayed under optimal conditions for phosphotransferase activity using 0.001 mg ml^−1^ of purified LpxT and 5 μM of Kdo_2_-[4′-^32^P]lipid A. As shown in Table 1, both ATP and IPP, which are precursors of C_55_-PP, assayed at 100 μM, did not stimulate phosphotransferase activity. Minor phosphotransferase activity was detected with FPP (100 μM), another precursor of C_55_-PP (Table 1), at 5000 times less than that seen with C_55_-PP.

**Table 1:** Phosphotransferase activity of purified LpxT with various phosphate donor substrates (100 μM).

Substrate	Specific activity^a^ (nmol min^−1^ mg^−1^)
ATP	Not detectable
DGPP	11.9
FPP	0.003
IPP	Not detectable
C_55_-P	Not detectable
C_55_-PP	13.7

a Kdo_2_-[4′-^32^P]lipid A (5 μM) was used as the acceptor substrate. Assays were performed within the linear range of time and enzyme concentration.

Conversely, DGPP (100 μM) served as an efficient donor substrate for LpxT in the phosphorylation of Kdo_2_-[4′-^32^P]lipid A (Table 1). DGPP contains a pyrophosphate group attached to diacylglycerol (Wu *et al*., 1996), and was previously shown *in vitro* to serve as a substrate for *E. coli* PgpB. Although DGPP served as an efficient phosphate donor for LpxT, there is no conclusive evidence that DGPP is found within the *E. coli* membrane (Dillon *et al*., 1996).

### *In vivo* formation of Lipid A 1-diphosphate is dependent upon the lipid a transporter, MsbA

C_55_-PP is synthesized on the cytoplasmic side of the inner membrane, but it is also regenerated on the periplasmic side of the inner membrane following various polymeriza tion reactions (e.g. polymerization of peptidoglycan) (Fig. 1). Dephosphorylation of C_55_-PP is required before it can be used for peptidoglycan biosynthesis (Fig. 1). Although several *E. coli* proteins showing UppP activity have been identified, it is still unclear how many phosphatases are involved in the metabolism of C_55_-PP and on what side of the inner membrane dephosphorylation of C_55_-PP occur.

**Fig. 7:**
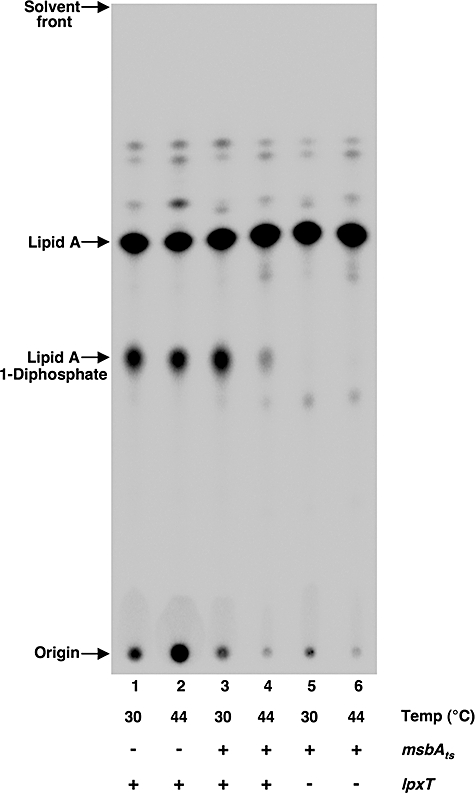
*In vivo* formation of lipid A 1-diphosphate is dependent upon MsbA. *E. coli* strains, BW25113A, BW2 and BW2 Δ*lpxT*, were temperature-shifted to 44°C for 30 min. Newly synthesized lipids were ^32^P-labelled for 20 min following the temperature shift as described previously (Tran *et al*., 2004), and the lipid A was isolated as described under *Experimental procedures*. Lipid A species from indicated strains were separated by TLC as described in the text and visualized by PhosphorImaging.

We have demonstrated that LpxT employs both C_55_-PP and Kdo_2_-lipid A as its substrates to form lipid A 1-diphosphate (Fig. 6B); however, we were uncertain if phosphorylation of lipid A takes place on the cytoplasmic or periplasmic side of the inner membrane. Previous work from our laboratory and that of others have shown that lipid A modifications generally occur after lipid A transport across the inner membrane by MsbA (Tran *et al*., 2004; Boon Hinckley *et al*., 2005; Wang *et al*., 2006a). MsbA-dependent transport of lipid A is lost in the *E. coli* temperature-sensitive mutant WD2 by shifting the cells from 30°C to 44°C for 30 min during mid-log phase (Doerrler *et al*., 2001). To determine whether the active site of LpxT is oriented towards the cytoplasmic or periplasmic surface of the inner membrane *in vivo*, derivatives of BW25113 harbouring a temperature-sensitive *msbA* mutation (*aroA::Tn10 msbA2*) (Doerrler *et al*., 2001) were constructed by P1_*vir*_ transduction (Table 2). These strains, designated BW2 and BW2 Δ*lpxT*, and the isogenic control strain BW25113A were grown at 30°C until the A_600_ reached 0.6–0.8. After 30 min at 44°C, the cells were labelled with 4 μC_i_ ml^−1^ of ^32^P_i_ for 20 min. The ^32^P-labelled lipid A species were isolated and separated by TLC. At 44°C, the control strain BW25113A synthesized the normal *E. coli* 1,4′-*bis-*phosphorylated lipid A and its 1-diphosphate derivative (Fig. 7, lane 2), whereas BW2 synthesized mainly the 1,4′-*bis-*phosphorylated lipid A species (lane 4). BW2 Δ*lpxT* at both temperatures synthesized only 1,4′-*bis-*phosphorylated lipid A (lanes 5 and 6). These results demonstrate that LpxT transfers a phosphate group from C_55_-PP to the lipid A domain of LPS within the periplasmic region of the cell. In support of our findings, Tatar *et al*. (2007) recently demonstrated by analysis of PhoA and GFP fusion proteins that the conserved acid phosphatase motifs of YbjG and LpxT (YeiU) face the periplasmic region of the cell.

**Table 2:** Bacterial strains and plasmids used in this study.

Strain	Genotype or description	Source or reference
CD43(DE3)		Avidis
W3110	Wild type, F^-^, λ^-^	*E. coli* Genetic Stock Center (Yale)
W3110A	Wild type, F^-^, λ^-^, *aroA::Tn10*	Doerrler *et al*. (2001)
WD2	W3110, *aroA::Tn10 msbA* (A270T)	Doerrler *et al*. (2001)
BW25113	*lacI*^*q*^*rrnB*_*T14*_Δ*lacZ*_*WJ16*_*hsdR514*Δ*araBAD*_*AH33*_Δ*rhaBADL*_*D78*_	Datsenko and Wanner (2000)
BW25113A	BW25113 *aroA::Tn10*	This work
BW2	BW25113 *aroA::Tn10 msbA* (A270T)	This work
DMEG1	BW25113 Δ*bacA*::Cam^R^	El Ghachi *et al*. (2005)
DMEG2	BW25113 Δ*ybjG*::Cam^R^	El Ghachi *et al*. (2005)
DMEG3	BW25113 Δ*lpxT*::Cam^R^	El Ghachi *et al*. (2005)
DMEG4	BW25113 Δ*pgpB*::Kan^R^	El Ghachi *et al*. (2005)
DMEG3/p*Trc*LpxT	BW25113 Δ*lpxT*::Cam^R^/p*Trc*LpxT	This work
DMEG3/p*Trc*LpxT-EHEC	BW25113 Δ*lpxT*::Cam^R^/p*Trc*LpxT-EHEC	This work
DMEG3/p*Trc*LpxT-ST	BW25113 Δ*lpxT*::Cam^R^/p*Trc*LpxT-ST	This work
CD43/pLpxTHIS	CD43(DE3)/pLpxTHIS	This work
Plasmids		
p*Trc*99A	Expression Vector containing a T7 promoter, Amp^R^	Amersham
p*Trc*LpxTEC	p*Trc*99A containing *lpxT*	El Ghachi *et al*. (2005)
p*Trc*LpxTEHEC	p*Trc*99A containing *lpxT* of *E. coli* O157:H7	This work
p*Trc*LpxTST	p*Trc*99A containing *lpxT* of *S. typhimurium LT2*	This work
pET2130	Derivative of pET21d (Novagen) – expression vector, Amp^R^	El Ghachi *et al*. (2004)
pLpxTHIS	pET2130 containing *lpxT*	This work

Cam^R^ and Kan^R^ represent inserted resistance genes to chloramphenicol and kanamycin respectively.

### Sequestering C_55_-PP with bacitracin prevents lipid A 1-diphosphate formation

**Fig. 8:**
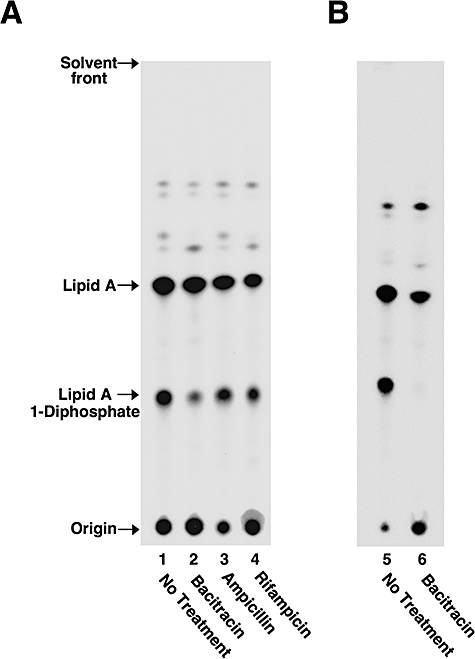
Bacitracin reduces formation of lipid A 1-diphosphate in whole cells. A. *E. coli* K-12 strain, BW25113, was exposed to various antibiotics at levels just below the MIC and labelled immediately with 2.5 μC_i_ ml^−1^ of ^32^P_i_ for 2.5 h. The antibiotic concentrations were as follows: ampicillin (10 μg ml^−1^), rifampicin (40 μg ml^−1^) and bacitracin (60 units ml^−1^). B. Complete inhibition of lipid A 1-diphosphate formation with high concentrations of bacitracin. *E. coli* K-12 strain, BW25113, was exposed to 150 units ml^−1^ of bacitracin for 50 min prior to labelling the cells with ^32^P_i_ for 20 min. ^32^P-labelled lipid A species from (A) and (B) were isolated as described under *Experimental procedures* and visualized by PhosphorImaging.

The decapeptide antibiotic bacitracin interdicts the recycling of the undecaprenyl carrier lipid by binding to the pyrophosphate domain of C_55_-PP (Storm and Strominger, 1973). To determine whether depletion of C_55_-PP in whole bacteria with bacitracin would prevent lipid A 1-diphosphate formation, *E. coli* wild-type strain BW25113 was grown at 37°C until the A_600_ reached ∼0.1. The cells were exposed to various antibiotics, as indicated, at levels just below the MIC, and labelled immediately with 2.5 μC_i_ ml^−1^ of ^32^P_i_ for 2.5 h. The ^32^P-labelled lipid A species were isolated and separated by TLC. Cells grown in the absence of antibiotics synthesized both lipid A and lipid A 1-diphosphate (Fig. 8A, lane 1). Similar results were seen when cells were exposed to ampicillin (10 μg ml^−1^), a β-lactam antibiotic that inhibits the formation of peptidoglycan cross-links in the bacterial cell wall, and to rifampicin (40 μg ml^−1^), an antibiotic that prevents transcription of messenger RNA (Fig. 8, lanes 3 and 4). However, cells exposed to bacitracin (60 units ml^−1^) displayed a reduction in the level of lipid A 1-diphosphate formation (lane 2) when compared with cells grown in the absence of any antibiotics (lane 1).

To examine if we can further reduce the formation of lipid A 1-diphosphate with bacitracin, cells were exposed to 150 units ml^−1^ of bacitracin for 50 min prior to the addition of ^32^P_i_. Presumably, this allowed for the complete sequestering of free C_55_-PP within the cell prior to pulse labelling with ^32^P_i_. As shown in Fig. 8B, depleting the available pool of free C_55_-PP completely prevented the formation of lipid A 1-diphosphate. These results clearly demonstrate that, *in vivo*, LpxT phosphotransferase activity is dependent upon C_55_-PP. Given there are approximately 10^6^ lipid A molecules within an *E. coli* cell with one-third of these residues modified with an additional phosphate (Raetz *et al*., 2007), LpxT must contribute significantly to the available pool of C_55_-P.

## Discussion

Both Gram-negative and Gram-positive bacteria have lipid-linked intermediary stages in their biosynthesis of various cell wall polysaccharides that are dependent on the carrier lipid C_55_-P (Wright *et al*., 1967; Scher *et al*., 1968; Watkinson *et al*., 1971; Johnson and Wilson, 1977; Rick *et al*., 1998; van Heijenoort, 2001b; Raetz and Whitfield, 2002). For example, in peptidoglycan biosynthesis, C_55_-P is required for the synthesis and transport of the hydrophilic GlcNAc-MurNAc-peptide monomeric motifs across the cytoplasmic membrane to the external sites of polymerization (Fig. 1). Another example is that C_55_-P can serve as acceptor for oligosaccharides repeat units as in the biosynthesis of O-antigen (Whitfield, 1995). Biosynthesis of C_55_-P is initiated from the dephosphorylation of C_55_-PP either on the cytosolic face of the inner membrane or on the periplasmic side during the late polymerization steps of peptidoglycan biosynthesis (Fig. 1) (van Heijenoort, 2001a).

Recently, several inner membrane proteins, BacA, PgpB, YbjG and YeiU, were identified in *E. coli* as having UppP activity (El Ghachi *et al*., 2004; 2005). Overexpression of these proteins in *E. coli* K-12 results in increased resistance to the cyclic polypeptide bacitracin, presumably by directly competing with the antibiotic for the available pool of free C_55_-PP. Multiple UppPs are thought to be involved in the metabolism of C_55_-P, because inactivation of at least three UppPs (BacA, YbjG and PgpB) enzymes are required to block cell wall synthesis and provoke cell lysis (El Ghachi *et al*., 2005). Interestingly, only one of the following genes, *bacA*, *ybjG* or *pgpB*, is necessary to support the growth of a temperature-sensitive UppP triple mutant (El Ghachi *et al*., 2005). Although YeiU was shown to exhibit UppP activity *in vitro*, inactivation experiments (El Ghachi *et al*., 2005) demonstrated that *yeiU* alone was not able to sustain growth of the temperature-sensitive UppP triple mutant. This result suggests that YeiU may have another role besides dephosphorylating C_55_-PP for peptidoglycan biosynthesis.

Lipid A isolated from wild-type *E. coli* K-12 is typically a hexa-acylated disaccharide of glucosamine with a monophosphate unit at positions 1 and 4′ (lipid A). However, one-third of the lipid A also contains an unsubstituted diphosphate unit at the 1 position (lipid A 1-diphosphate). All of the enzymes required for making lipid A in *E. coli* K-12 have been described, with the exception of the reaction that generates the 1-diphosphate unit. We now provide concrete evidence that YeiU (renamed LpxT) is responsible for the 1-diphosphate moiety found at the C-1 position of *E. coli* K-12 lipid A. Mutants lacking a functional copy of *lpxT* were deficient in synthesizing lipid A 1-diphosphate (Fig. 3). Using an *in vitro* assay, we determined that membranes of *E. coli* K-12 *lpxT*-deficient mutant were unable to convert Kdo_2_-lipid A to Kdo_2_-lipid A 1-diphosphate (Fig. 4B).

Our data demonstrate that LpxT specifically uses the carrier lipid C_55_-PP for phosphotransferase activity (Fig. 5), and that formation of lipid A 1-diphosphate *in vivo* is dependent upon the transfer of lipid A across the inner membrane by MsbA (Fig. 6). The fact that LpxT requires MsbA *in vivo* provides compelling evidence to support the periplasmic orientation (Tatar *et al*., 2007) of its active site (Fig. 1). This work provides the first biochemical evidence that dephosphorylation of C_55_-PP occurs on the periplasmic side of the inner membrane, and also corroborates the UppP activity of LpxT previously reported by El Ghachi *et al*. (2005). However, it is yet to be determined on which side of the inner membrane dephosphorylation of C_55_-PP occurs with the other UppPs during peptidoglycan biosynthesis.

Modification of negatively charged phosphate groups of lipid A with positively charged amine-containing substituents is an important strategy employed by a wide variety of Gram-negative bacteria to increase resistance to cationic antimicrobial peptides (Trent *et al*., 2006; Raetz *et al*., 2007). In some organisms, these modifications are under the control of the PhoP/PhoQ and the PmrA/PmrB two-component regulatory systems (Guo *et al*., 1997; Gunn *et al*., 1998). Pathogenic *E. coli* or *E. coli* K12 in which PmrA is constitutively active produces lipid A species modified with pEtN and/or L-Ara4N (Zhou *et al*., 1999; Kim *et al*., 2006), but not species containing the 1-diphosphate moiety. However, expression of LpxT from either *E. coli* O157:H7 or *S. typhimurium* LT2 can successfully complement an *E. coli* K-12 *lpxT* mutant (Fig. 5). Additionally, it has been shown that *Salmonella pmrA*-null mutants that are unable to modify their lipid A with pEtN or L-Ara4N synthesize the 1-diphosphate species (Zhou *et al*., 2001). Taken together, these data suggest that PmrA may play a role in the regulation of the 1-diphosphate species. Further studies are under way to determine if this regulation is occurring at the transcriptional level of *lpxT*, or possibly at the post-translational level within the bacterial membrane.

This work also provides another example of how undecaprenyl carrier lipids are not only involved in the biosynthesis of the O-antigen domain of LPS, but also in the modification of its hydrophobic anchor. For example, the periplasmic modification of lipid A by the aminoarabinose transferase requires undecaprenyl-phospho-L-Ara4N (Trent *et al*., 2001b). Similarly, the attachment of galacturonic acid residues to the lipid A and core domains of *Rhizobium leguminosarum* LPS (Kanjilal-Kolar and Raetz, 2006), and the incorporation of galactosamine units into lipid A in *Francisella tularensis* subsp. *novicida* (Wang *et al*., 2006b) are dependent upon undecaprenyl-linked intermediates. The biological function of the diphosphate moiety on lipid A in *E. coli* K-12 is still unclear as LpxT was not essential for cell growth on nutrient broth. Of interest was that loss of LpxT function resulted in the formation of a minor lipid A species that resembled the addition of a single pEtN unit (Fig. 3A). The presence of a diphosphate group or pyrophosphoethanolamine in the lipid A structure may provide some benefit to the bacterium by increasing the stability of the outer membrane under different environmental conditions. Perhaps the diphosphate moieties of lipid A are used as an energy source within the extracytoplasmic compartment of the bacterial cell as ATP is not available. Given that LpxT function is directly linked to the synthesis of C_55_-P, and that modification of the lipid A domain of LPS has been shown important for bacterial pathogenesis (Guo *et al*., 1997; van Velkinburgh and Gunn, 1999; Gunn *et al*., 2000; Gunn, 2001; Gibbons *et al*., 2005), further investigation of the function and regulation of LpxT is warranted.

## Experimental procedures

### Chemicals and other materials

[γ-^32^P]ATP and ^32^P_i_ were obtained from GE Healthcare Bio-Science AB. [^14^C]C_55_-PP was from Perkin Elmer. DDM was purchased from Anatrace. Silica gel 60 (0.25 mm) thin-layer plates were purchased from EM Separation Technology (Merck). Yeast extract and tryptone were from Difco. Triton X-100 and bicinchoninic acid (BCA) were from Pierce. ATP, IPP, FPP and bacitracin were purchased from Sigma. DGPP was obtained from Avanti Polar Lipids. C_55_-P and C_55_-PP were purchased from Institute of Biochemistry and Biophysics, Polish Academy of Sciences.

### Bacterial strains and growth conditions

Bacterial strains and plasmids are summarized in Table 2. *E. coli* strain BW25113 harbouring mutations in genes encoding enzymes with C_55_-PP phosphatase activity were previously constructed (El Ghachi *et al*., 2004; 2005). To construct a strain of BW25113 harbouring a temperature-sensitive mutation [*aroA*::*Tn10 msbA* (A270T)] that results in loss of MsbA function at 44°C, a P1_vir_ lysate of WD2 was used to transduce BW25113 to tetracycline resistance as described previously (Miller, 1972). Resulting colonies were repurified and tested for temperature sensitivity to give the strain BW2. The temperature-sensitive mutation was also introduced into the *lpxT* mutant of BW25113. A marker derivative of BW25113, designated BW25113A, was also prepared by transduction using a P1_vir_ lysate of W3110A (see Table 2). Bacteria were routinely grown at 37°C in Luria–Bertani (LB) broth or on LB agar unless indicated otherwise. Cultures were supplemented with ampicillin (100 μg ml^−1^), chloramphenicol (30 μg ml^−1^) and/or kanamycin (30 μg ml^−1^) when appropriate.

### Recombinant DNA techniques

Plasmid isolation, PCR clean-up and DNA gel-isolation kits were performed as per manufacturer's instructions (Qiagen). Custom primers were obtained from Integrated DNA Technologies. PCR reagents were purchased from Stratagene. Restriction endonucleases, T4 DNA ligase and antarctic phosphatase were purchased from New England Biolabs.

### Isolation and analysis of lipid A and phospholipids species from ^32^P_i_-labelled cells

Typically, a 7.5 ml cell culture was labelled uniformly with 2.5 μCi ml^−1 32^P_i_ until the cells reached late-log phase. Bacteria were collected using a clinical centrifuge, and washed with 5 ml of phosphate-buffered saline (pH 7.4). ^32^P-labelled lipid A and phospholipids were isolated using published protocols (Zhou *et al*., 1999) and spotted onto a Silica Gel 60 TLC plate (∼10 000 c.p.m. per lane). Lipids were separated using the solvent chloroform, pyridine, 88% formic acid, water (50:50:16:5, v/v), and visualized using a Bio-Rad Molecular Imager PhosphoImager equipped with Quantity One software.

### Isolation of lipid A ^32^P_i_-labelled cells exposed to antibiotics

Bacteria were grown at 37°C until an A_600_ of ∼0.1 was reached. Cells were then exposed separately to MICs of the following antibiotics: bacitracin (60 units ml^−1^), ampicillin (10 μg ml^−1^) or rifampicin (40 μg ml^−1^). ^32^P_i_ at 2.5 μC_i_ ml^−1^ was added to the growth media and the labelling continued for 2.5 h. To completely sequester C_55_-PP within whole cells, bacteria were grown as described above, and then exposed to bacitracin (150 units ml^−1^) for 50 min prior to the addition of 2.5 μCi ml^−1^ of ^32^P_i_. As this level of bacitracin was lethal to the bacteria, cells were only pulsed labelled for 20 min. The ^32^P-labelled lipid A species were isolated and separated by TLC as described above.

### Construction of plasmids

The plasmid p*Trc*LpxT allowing expression of the *lpxT* gene under control of the isopropyl-β-D-thiogalactopyranoside (IPTG)-dependent *trc* promoter was generated as previously described (El Ghachi *et al*., 2005). The *lpxT* gene was amplified by PCR from *E. coli* O157:H7 and *S. typhimurium* LT2 genomic DNA using the following oligonucleotides: U-EHECLpxT and L-EHECLpxT, and U-STLpxT and L-STLpxT (Table 3) respectively. The resulting fragments were digested with BspHI and HindIII, gel-purified and cloned between the NcoI and HindIII sites of p*Trc*99A to generate the following plasmids: p*Trc*LpxT-EHEC and p*Trc*LpxT-ST. A His-tagged version of LpxT was constructed by using the following primers: LpxTBamHI and LpxTHindIII (Table 3), to PCR-amplify the gene *lpxT* from *E. coli* genomic DNA. The PCR product, engineered to contain a BamHI and a HindIII sites at the 5′ and at the 3′ end of the coding region respectively, was cloned into the corresponding sites of the T7 expression vector pET2130 (El Ghachi *et al*., 2004). The resulting plasmid, pLpxTHis, allowed the expression of a N-terminally His_6_-tagged form of LpxT protein under the control of the strong IPTG-inducible T7 promoter.

**Table 3:** Oligonucleotides.

Name	Sequence
LpxT1	5′-GAAATCATGATTAAAAATTTGCCGCAAATAGTGTTGTTG-3′
LpxT2	5′-ATGAAAGCTTGGTGCGCATCATCAGGATTATCCTCAC-3′
U-EHECLpxT	5′-GCGCGCTCATGATTAAAAATTTGCCGCAAA-3′
L-EHECLpxT	5′-GCGCGCAAGCTTTTATTTGTTTTGGAAATG-3′
U-STLT2LpxT	5′-GCGCGCTCATGACGATGAAAACCCGCTATT-3′
L-STLT2LpxT	5′-GCGCGCAAGCTTTTATTTGTTTAAAATTTG-3′
LpxTBamHI	5′-GCGCGGATCCATGATTAAAAATTTGCCGCAAATAG-3′
LpxTHindIII	5′-GCGCATCATCAAGCTTATCCTCACTATTTT-3′

### Expression and purification of LpxT

*Escherichia coli* C43(DE3) (Avidis) cells carrying the plasmid pLpxTHis were grown at 37°C in 2× tryptone-yeast extract medium (1 l) containing 100 mg ml^−1^ of ampicillin. When the optical density (A_600_) of the culture reached 1.0, IPTG was added at the final concentration of 1 mM, and the growth was continued for 3 h. Cells were then harvested (4000 *g*, 10 min) and resuspended in 40 ml of 20 mM Tris-HCl, pH 7.5, 1 mM MgCl_2_, 20 mM β-mercaptoethanol, 0.5 M NaCl and 15% glycerol (buffer A). Cells were disrupted by three successive passages through a French press; membranes and soluble proteins were segregated by ultracentrifugation (100 000 *g*, 1 h). The resulting pellet containing membrane proteins was washed three times in 20 ml of buffer A. The membrane proteins were solubilized by incubation in 20 ml of buffer A supplemented with 2% (w/v) DDM (buffer B) for 2 h at 4°C. The solution was centrifuged (100 000 *g*, 1 h), and the supernatant was incubated with 2 ml of nickel-nitrilotriacetate-agarose (Ni^2+^-NTA) and 10 mM imidazole at 4°C overnight. The resin was washed successively with 20 vols of 10 mM, 20 mM and 30 mM imidazole solutions prepared in buffer B, and the proteins were eluted with buffer B supplemented with 400 mM imidazole. The purified protein at 0.5 mg ml^−1^ was either stored in state at −20°C, or thoroughly dialysed against buffer A supplemented with 0.04% DDM before being stored at −20°C. In the latter case, more than 50% of the protein was lost as a result of precipitation. Protein concentration was determined by the BCA method (Smith *et al*., 1985), using bovine serum albumin as the standard.

### Preparation of cell-free extracts, double-spun cytosol and washed membrane

Typically, 200 ml of *E. coli* cultures was grown to an A_600_ of 1.0 at 37°C and harvested by centrifugation at 10 000 *g* for 10 min. All samples were prepared at 4°C. Cell-free extract, double-spun cytosol and washed membranes were prepared as previously described (Trent *et al*., 2001c), and were stored in aliquots at −20°C. Protein concentration was determined by the BCA method.

### Undecaprenyl pyrophosphate phosphatase assay

The radiolabelled [^14^C]C_55_-PP substrate was prepared as previously described (El Ghachi *et al*., 2004) by successive condensations of [^14^C]IPP to FPP (Sigma) catalysed by the purified UppS enzyme. The C_55_-PP phosphatase assay was performed in a 20 μl reaction mixture containing 20 mM Tris-HCl, pH 7.5, 20 mM MgCl_2_, 10 mM β-mercaptoethanol, 150 mM NaCl, 0.6% DDM, 100 μM [^14^C]C_55_-PP (2305 Bq) and purified LpxT enzyme. In order to examine the effect of lipid A on C_55_-PP phosphatase activity of LpxT, purified lipid A was added to the reaction mixture. The reaction mixture was incubated 30 min at 37°C, and terminated by heating at 100°C for 1 min. Assays were performed within the linear range of time and enzyme concentration. The sample was spotted onto a Silica Gel 60 TLC plate, and the substrate (C_55_-PP) and reaction product (C_55_-P) separated using the solvent di-isobutyl ketone, acetic acid and water (8:5:1, v/v) as a mobile phase (R*f* values of C_55_-PP and C_55_-P were 0.36 and 0.5 respectively). The radioactive spots were located and quantified with a radioactivity scanner (model Multi-Tracermaster LB285; Berthold-France).

### Assay of phosphate transfer to Kdo_2_-lipid A

The substrate Kdo_2_-[4′-^32^P]lipid A was synthesized as previously described (Tran *et al*., 2004). The 1-phosphotransferase activity of purified LpxT (0.001 mg ml^−1^) was assayed under optimized conditions in a 10 μl reaction mixture containing 50 mM HEPES, pH 7.0, 0.3% Triton X-100, 5 μM Kdo_2_-[4′-^32^P]lipid A (∼5000 c.p.m. nmol^−1^) and 100 μM of C_55_-PP as the donor substrate. For comparison, phosphate transfer to the Kdo_2_-[4′-^32^P]lipid A acceptor was also tested with the phosphate donors (100 μM) listed in Table 1. When washed membranes (0.5 mg ml^−1^) were employed as the enzyme source, assays contained 1.0% Triton X-100. Phosphotransferase reactions were incubated at 30°C for 180 min and terminated by spotting 4.5 μl portions of the mixtures onto silica gel 60 TLC plates. Reaction products were separated using the solvent chloroform, pyridine, 88% formic acid, water (30:70:16:10, v/v), and visualized as described above. The enzyme activity was calculated by determining the percentage of the substrate converted to product.

## Supplementary material

This material is available as part of the online article from:

http://www.blackwell-synergy.com/doi/abs/10.1111/j.1365-2958.2007.06044.x

(This link will take you to the article abstract).
