# Supplementary material for: Periplasmic phosphorylation of lipid A is linked to the synthesis of undecaprenyl phosphate
Source: Mol Microbiol. 2007 Nov 2;67(2):264–77. doi: 10.1111/j.1365-2958.2007.06044.x (PMC2229476; doi:10.1111/j.1365-2958.2007.06044.x)
Supplement: Supplementary file 1 [file mmi0067-0264-SD1.pdf]

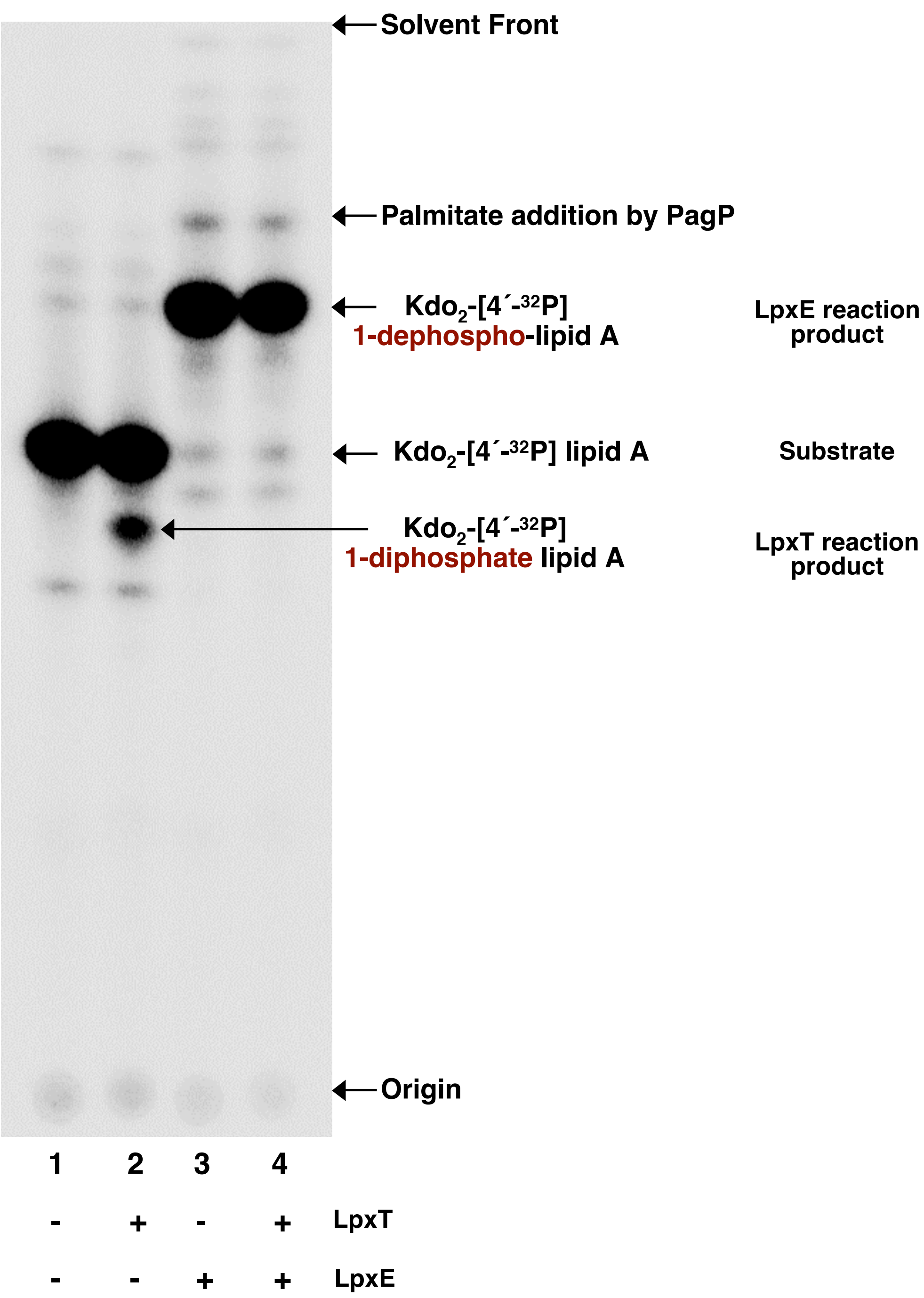

Confirmation of phosphate transfer to the 1-posiiton of *E. coli* Kdo$_2$-lipid A. Pure LpxT (0.001 mg/ml) was used to generate the 1-diphosphate reaction product using Kdo$_2$-[4′-^{32}P]lipid A and C$_{55}$-PP for one hour. Following phosphate transfer, the reaction mixture was treated with *E. coli* membranes over expressing the lipid A 1-phosphatase, LpxE, for an additional hour at 30°C. The reaction products were separated by TLC and detected with PhosphorImager analysis. Convergence of both the reaction product (1-diphosphate) and the initial substrate to a single TLC spot indicated that LpxT-dependent phosphorylation occurrs at the 1-position.
